# Influence of Ultraviolet/Ozonolysis Treatment of Nanocarbon Filler on the Electrical Resistivity of Epoxy Composites

**DOI:** 10.1186/s11671-016-1577-4

**Published:** 2016-08-22

**Authors:** Yulia Perets, Lyudmila Matzui, Lyudmila Vovchenko, Irina Ovsiienko, Olena Yakovenko, Oleksandra Lazarenko, Alexander Zhuravkov, Oleksii Brusylovets

**Affiliations:** 1 Physics Department, Taras Shevchenko National University of Kyiv, 64/13 Volodymyrska Str., Kyiv, 01601 Ukraine; 2 Chemistry Department, Taras Shevchenko National University of Kyiv, 64/13 Volodymyrska Str., Kyiv, 01601 Ukraine

**Keywords:** Polymer composite, Graphite nanoplatelets, Ultraviolet treatment, Percolation threshold, Electrical conductivity, 72.80.Tm, 64.60.ah, 82.35.Np

## Abstract

In the present work, we have investigated concentration and temperature dependences of electrical conductivity of graphite nanoplatelets/epoxy resin composites. The content of nanocarbon filler is varied from 0.01 to 0.05 volume fraction. Before incorporation into the epoxy resin, the graphite nanoplatelets were subjected to ultraviolet ozone treatment at 20-min ultraviolet exposure. The electric resistance of the samples was measured by two- or four-probe method and teraohmmeter E6-13. Several characterization techniques were employed to identify the mechanisms behind the improvements in the electrical properties, including SEM and FTIR spectrum analysis.

It is established that the changes of the relative intensities of the bands in FTIR spectra indicate the destruction of the carboxyl group –COOH and group –OH. Electrical conductivity of composites has percolation character and graphite nanoplatelets (ultraviolet ozone treatment for 20 min) addition which leads to a decrease of percolation threshold 0.005 volume fraction and increase values of electrical conductivity (by 2–3 orders of magnitude) above the percolation threshold in comparison with composite materials—graphite nanoplatelets/epoxy resin. The changes of the value and behavior of temperature dependences of the electrical resistivity of epoxy composites with ultraviolet/ozone-treated graphite nanoparticles have been analyzed within the model of effective electrical conductivity. The model takes into account the own electrical conductivity of the filler and the value of contact electric resistance between the filler particles of the formation of continuous conductive pathways.

## Background

Progress in various fields of science and technology provides the creation of new materials with the required properties. These materials put high demands on strength, hardness, conductivity, heat resistance, and so on. Most of these requirements are satisfied in the industry due to the wide usage of polymer composite materials (CMs). The composite materials with carbon fillers such as graphite, carbon nanotubes, carbon fibers, carbon black, and graphite nanoplatelets (GNPs) are very popular today [1].

As it is known, graphite has excellent electrical [2] and thermal conductivity [3, 4] due to its layered structure and has unique mechanical properties, with very high modulus along its graphene plane. These useful properties combined with very low cost, especially compared to carbon nanotubes, make it a popular filler to produce conducting polymers for applications in areas such as electromagnetic interference shields [5, 6] and thermal conductors.

The graphite nanoplatelets are produced as a result of the thermochemical treatment of the natural disperse graphite (surface oxidation and thermal shock—get thermally expanded graphite (TEG)) and ultrasonic dispersing (UD) of thermally expanded graphite in various fluid environments [7–9]. One of the most widely used methods of nanoparticle surface oxidation is the treatment by strong acids such as HNO_3_ [10] and H_2_SO_4_ [11].

Using acids as liquid processing agents is expected to result in a high degree of nanocarbon dispersion due to the formation of strong covalent bonds between functional groups and carbon atoms. In this case, however, the delocalized π-electronic system of graphite layer is destructed and σ-bonds are partially broke, while free bonds that are formed provide the attachment of various functional groups to the nanocarbon surface. At the same time, chemical functionalization by strong acids results in the formation of a large quantity of defects on the nanocarbon surface [12].

Consequently, the splitting of source micron graphite particles into individual particles 5–65 nm thick (GNPs) as well as their functionalization with different functional groups and formation of defects on the nanocarbon surface occurred depending on the type of liquid reagent for ultrasonic dispersing.

One of the most popular methods of cleaning the surface nanocarbon after functionalization is the ultraviolet (UV) ozone treatment (or UV/O_3_), which is fast and safe.

Ultraviolet treatment of nanocarbon with small doses leads to strengthening (due to additional functionalization) of connection between nanocarbon fillers and the polymer matrix at interfaces [13] and can lead to increased efficiency of chemical interactions between the surface of nanocarbon and polymer matrix in composites [14–16], thus improving the electrical, thermal, and mechanical properties in polymer composites [17]. However, the concentration and temperature dependences of the electrical conductivity of the composites with ultraviolet ozone-treated graphite nanoparticles are not sufficiently investigated. The issue of the influence of ultraviolet ozone-treated nanocarbon on percolation characteristics in polymer composites was not yet disclosed.

The aim of this study was to investigate the effect of ultraviolet ozone treatment of carbon filler on the concentration and temperature dependences of the electrical conductivity of polymer composite materials epoxy/graphite nanoplatelets in order to enhance the conductivity of composite materials with low content of nanocarbon filler.

## Methods

### Measurements

The morphology of natural disperse graphite, expanded graphite (TEG), and GNPs was examined by scanning electron microscope (SEM; Mira3 Tescan) at accelerating voltage of 10.0 kV.

Ultrasonic dispersion of TEG powder occurs in ultrasonic bath “BAKU” BK-9050, ultrasonic frequency—40 kHz, with a maximum electrical power output of 30 and 50 W.

UV ozone treatment was performed by DRT-1000 (ultraviolet lamp) equipped with electric-discharge arc lamp of high pressure inflated with mercury and argon compound that could release ultraviolet radiation of 50 W at 240–320 nm wavelength. The distance between the UV lamp and the sample was fixed at 11 cm.

The IR spectra were obtained by PerkinElmer Spectrum BX FT-IR infrared spectrometer in the frequency range 4000–400 cm^−1^ in transmission mode for TEG and GNPs before and after ultraviolet ozone treatment.

The electric resistance of the samples was measured by two-probe (*R* = 10^4^–10^9^ Ω) and four-probe (*R* ≤ 10^4^Ω) method or teraohmmeter E6-13 (*R* = 10^9^–10^13^ Ω). An automated installation was used for the investigation of the temperature dependence of the electrical resistance in the temperature range of 6–300 K. The main components of automated installation are as follows: a rod for a sample, a power-switching current direction and a stable source of voltage, an analog-digital converter ADC 16–32F (SDI), a personal computer, and the interface cables. The temperature was measured by a copper-constantan thermocouple located near the sample. The measurement range of electric resistance (0.01–10^14^ Ω) was divided into several regions: 0.01–2.5 Ω, where error does not exceed 0.5 %; 2.5–10^7^ Ω (error was < 1 %); *R* = 10^8^ Ω (< 5 %); *R* = 10^9^ Ω (< 10 %); and *R* = 10^10^–10^13^ Ω (<20 %). When measuring the electric resistance of CMs, three samples for each concentration were tested. The scalar network analyzer was used to measure the transmission and reflection loss within the 25.5–37.5-GHz frequency range.

### Materials

**Fig. 1:**
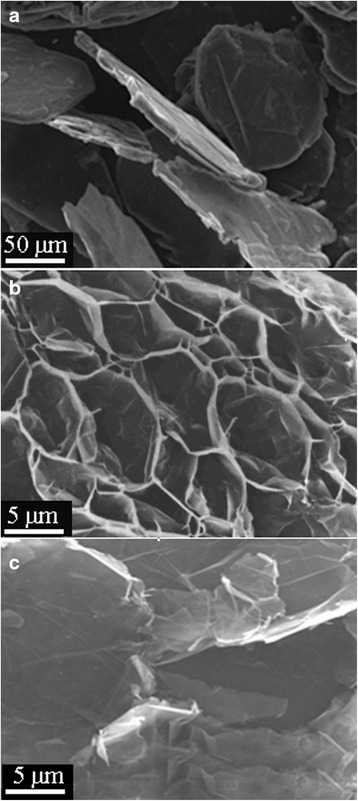
SEM images. **a** Natural disperse graphite. **b** TEG. **c** TEG after 3 h of ultrasonic dispersing in acetone (GNPs)

The initial material for graphite nanoplatelets obtained was thermally expanded graphite (*d* = 50–100 μm, *l* = 3–5 mm). TEG is a product of natural disperse graphite (*d* = 50–300 μm, *h* = 5–30 μm) intercalation with H_2_SO_4_ and subsequent heat treatment in a furnace with ascending flow according to the method developed, and it was reported in a previously published paper (Fig. 1). To break the obtained graphite sheets into the individual or groups of GNPs, they were sonicated in an acetone medium for 3 h at 40 kHz and 50 W. The electron microscopy investigations have shown that obtained GNPs are disk-shaped particles. Their diameter is about 0.2–30 μm and their thickness is about 5–65 nm (Fig. 1c).

Then, the GNP powders were divided in two parts. One part was subjected to UV/ozone treatment, while the other was left intact. The initial GNP powders were subjected to UV/ozone treatment for 20 min (GNP (UV/O_3_)).

### Preparation of Composites

For the investigation of the concentration dependences of the conductivity, the GNP/polymer composites with 0.01–0.05 volume fraction (vol. fr.) of filler were prepared. Epoxy resin Larit285 (viscosity of 600–900 mPa s) with hardening agent H285 (viscosity of 50–100 mPa s) was used as the polymer matrix. UV-treated (*t* = 20 min) GNP filler is mixed with epoxy resin and acetone. A mixture of these components was stirred an additional 15 min in an ultrasonic bath for more uniform distribution of the filler in the polymer, then the curing agent H285 was added, and a mixture was poured into molds and cured at room temperature. To complete the polymerization, the molds with composite mixtures were exposed to the temperature that gradually increased from 40 to 80 °C for 5 h.

For the measurements of electrical conductivity, the samples with the shape of a rectangular parallelepiped with size 3.5 × 3.5 × 10 mm^3^ were prepared. The measuring conductivity range was from 10^−12^ to 10 S/m.

## Results and Discussion

### Ultraviolet/Ozonolysis Treatment of Nanocarbon

To verify the possibility of chemical functionalization as the result of UV/ozone treatment, the IR investigation of GNP before and after UV/ozone treatment has been done. Also, the IR spectrum of TEG sample was recorded.

**Fig. 2:**
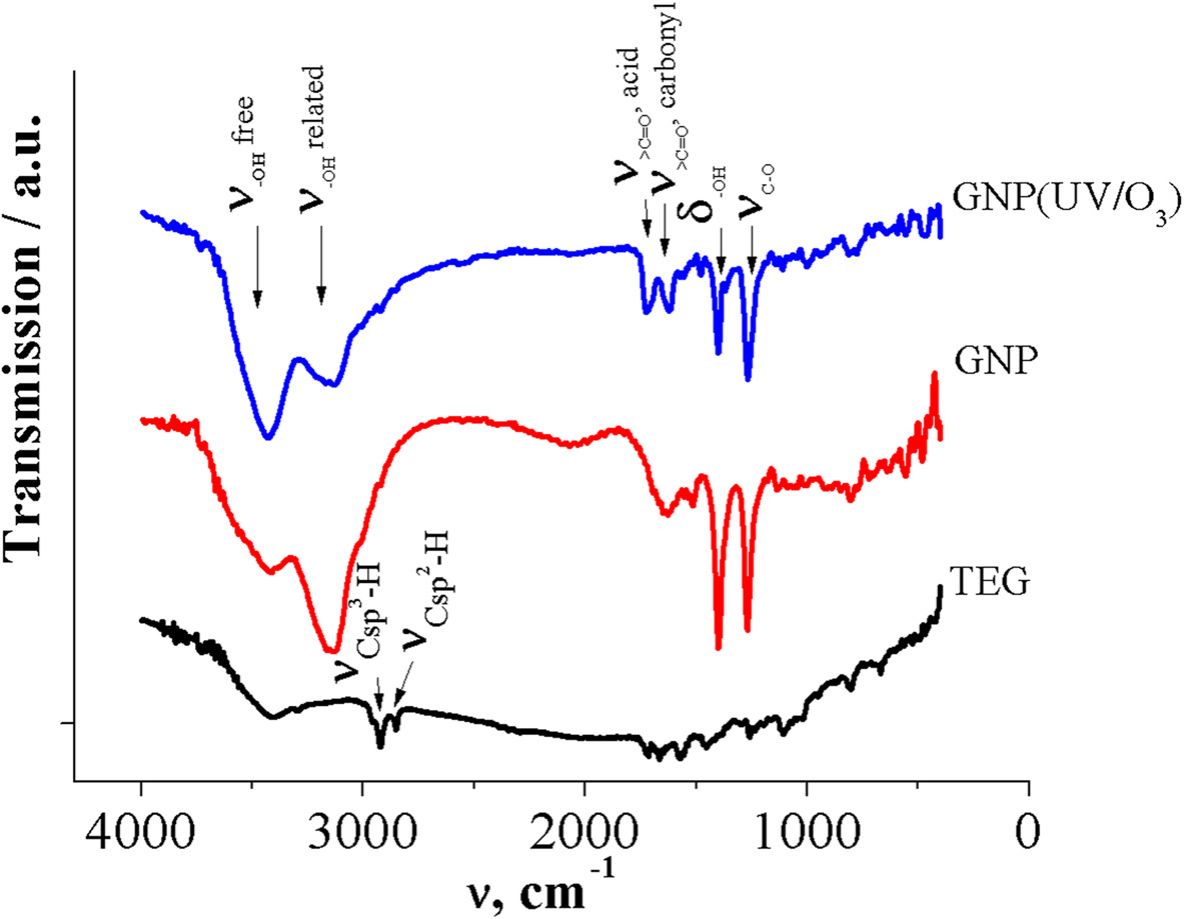
IR spectra of TEG and GNPs before and after UV/ozone treatment

Figure 2 presents the IR absorption spectrum for the source TEG. Evidently, the absorption spectrum for TEG contains a series of peaks and bands, two of which correspond to the impurities, namely, a wide band at 3410 cm^−1^ corresponding to the vibration of the bounded –OH groups (water). A group of peaks is observed in the IR spectrum of TEG in the range 3000 cm^−1^. They are related to the valence C–H vibrations. Besides, several peaks near 1600 cm^−1^ corresponding to the vibration of C=C and C=O multiple bonds are also observed.

Ultrasonic dispersion of TEG powder is carried out in a ultrasonic bath in a liquid medium of acetone for 3 h to obtain the GNPs. As a result, the particles are stratified along the TEG planes with van der Waals forces and we obtain particles of a micron size in plane and nanometer thickness.

As we can see from Fig. 2a, a number of intense lines which do not exist in the IR spectrum of TEG particles have appeared in the IR spectrum of GNPs: vibrations (δOH), vibrations of C–O group, and wide double-peak line in the band (3440–3160) cm^−1^ corresponds to fluctuations of hydroxyl group. Also, as it is seen from IR spectra in Fig. 2, the lines which corresponded to fluctuations of C_sp_^2^–H (2900 cm^−1^) and C_sp_^3^–H (2800 cm^−1^) groups and other lines observed in the spectrum of TEG are not practically observed for the initial sample of GNPs as well as for irradiated samples of GNPs. Such low relative intensity of the lines associated with fluctuations of carbon atoms is obviously explained by the high relative intensity of the lines that correspond to fluctuations of functional groups.

So, as we can see from Fig. 2, the functionalization of the surface of GNP particles has occurred under the ultrasonic dispersion of TEG in the acetone medium.

The UV/O_3_ treatment of GNP powder was carried out at atmospheric pressure.

The UV/O_3_ treatment is a photo-oxidation process, when the molecules are excited by the absorption of short-wavelength UV radiation, while the carbon atoms at the defect sites of nanotube surface may react with the atomic oxygen from the continuous dissociations of the oxygen molecules.

So, the additional functionalization of the surface of GNP particles could occur in this process. Simultaneously, the UV treatment leads to cleaning of the surface of the GNP particles. SEM investigation of GNP surfaces before and after UV treatment presented in [16] has shown that the UV-treated GNPs also exhibit a rougher surface as well as clearer boundaries between the individual basal planes in comparison with the untreated graphite. It indicates that loosely bonded materials and organic contaminants on the GNP surface were removed through the UV treatment.

IR spectrum of the initial GNP powder is characterized by a number of bands that correspond to different functional groups. Wide double-peak line in the band (3440–3160) cm^−1^ corresponds to fluctuations of hydroxyl group. After 20 min of UV/ozone treatment, the redistribution of the relative intensities of these two peaks is observed: the relative intensity of the band corresponded to the free –OH increases and the relative intensity of bands corresponded to –OH bound decreases. Perhaps such a redistribution of the relative intensities of the bands indicates the destruction of the carboxyl group COOH and of group –OH under UV irradiation.

Let us consider the band that corresponds to the vibrations of group >C=O. This band is formed by separate lines, which is part of the carboxyl and ketone groups (short plot) and carbonyl and lactones groups (long-wave area). Relative integrated intensity of this band for GNP (UV/O_3_) is increasing. In the spectra of GNPs, two bands identified as deformation vibrations (δOH) –OH group (1409 cm^−1^) and vibrations group of C–O (1263 cm^−1^) also are present. In the spectrum for GNP (UV/O_3_), the intensities of these bands are decreased.

Thus, as follows from the IR spectra, UV/O_3_ functionalized GNPs do not lead to the appearance of additional functional groups on the surface of the GNPs. The number of functional groups that arise on the surface of carbon due to UV/ozone treatment and functionalization ozone significantly is less as compared with the number of functional groups joining the carbon at the surface after chemical functionalization (acetone treatment). UV/ozone functionalization of carbon apparently causes partial destruction of some functional groups, such as, for example, carboxyl group, and also reduces the deformation vibrations (δOH) and vibrations of C–O group.

### Electrical Conductivity of the Polymer Composites with GNPs and GNP (UV/O_3_)

**Fig. 3:**
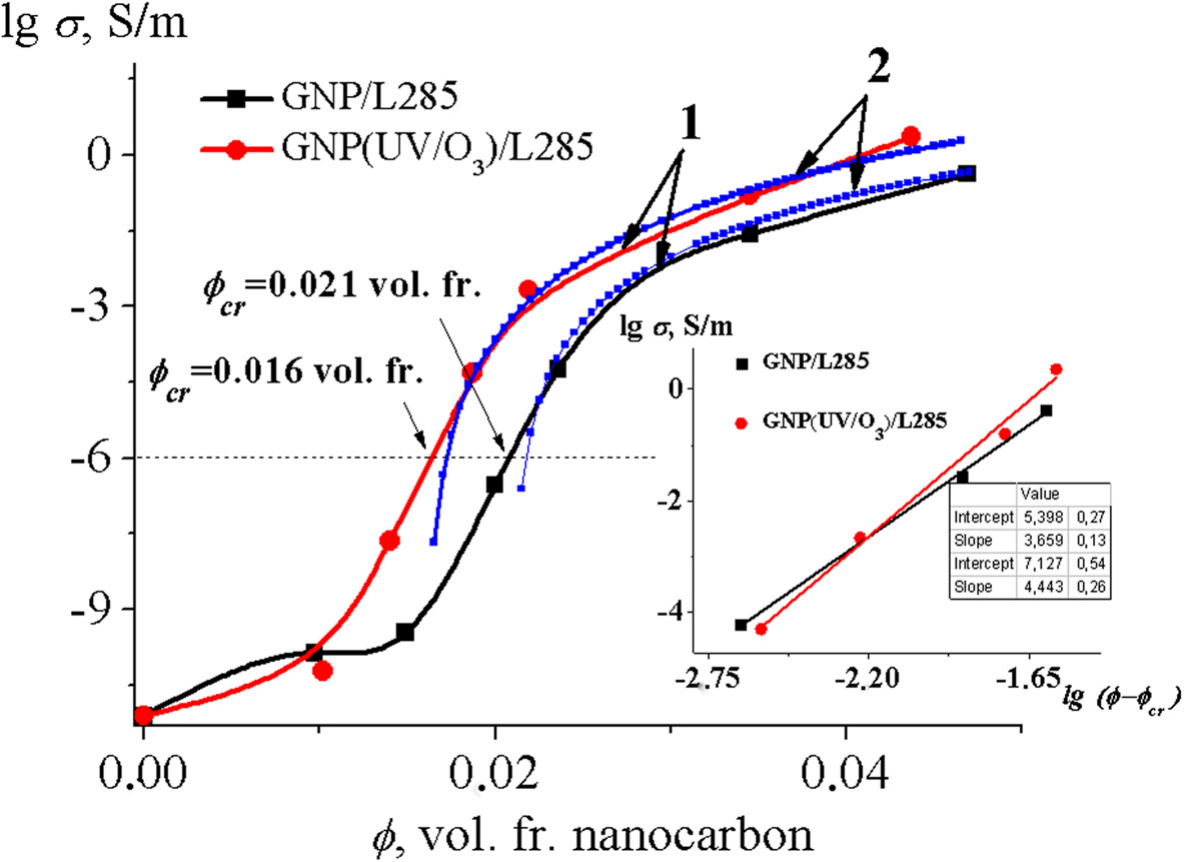
Concentration dependence of electrical conductivity of polymer composite materials with GNP and GNP (UV/O_3_). *Inset* shows a scaling dependence (lg *σ* as a function of lg *(ϕ − ϕ*_cr_) for the determining parameters of Eq. (1). 1 The experimental curves; 2 the calculated curves according to Eq. (1)

The electrical conductivity of CMs with nanocarbon filler (GNP/L285, GNP (UV/O_3_)/L285) was investigated. The concentration dependences of electrical conductivity of investigated CMs are presented in Fig. 3. As one can see, the electrical conductivity demonstrates the percolation behavior, and percolation threshold is observed at sufficiently low volume content of filler, *ϕ*_cr_ = 0.021 vol. fr. for CMs with GNPs. As it is seen from Fig. 1, for the composite materials GNP (UV/O_3_)/L285, percolation transition has shifted to a lower concentration range of 0.005 vol. fr. and *ϕ*_cr_ = 0.016 vol. fr. Also, there is a significant increase in the value of the electrical conductivity of GNP (UV/O_3_)/L285 composites for the same content of GNPs in a polymer matrix.

For example, for CM GNP/L285 with a concentration of 0.0236 vol. fr. GNPs, the electrical conductivity at room temperature is *σ =* 5.8 × 10^−5^ S/m, and for CM GNP (UV/O_3_)/L285, the same concentration conductivity is *σ =* 2.1 × 10^−3^ S/m, which is 36 times more.

It is known that the concentration dependence of the electrical conductivity of filled polymers is described by three parameters: the critical concentration *ϕ*_cr_ (percolation threshold), the critical index *t*, and conductivity of filler *σ*_filler_ in the classical percolation model:

1 $$ \sigma \left(\phi \right)\sim {\sigma}_{\mathrm{filler}}{\left(\phi -{\phi}_{\mathrm{cr}}\right)}^t $$

Analysis of *σ* (*ϕ*) dependencies for samples showed that they can be satisfactorily described by Relation (1). Using the scaling dependence *lgσ* ~ *lg(ϕ − ϕ*_cr_*)*, critical index *t*, and electrical conductivity related with filler *σ*_filler_ (inset in Fig. 3 and Table 1) were determined.

**Table 1:** Percolation characteristics of nanocarbon polymer CMs with GNP and with GNP (UV/O_3_)

Nanocomposite	*ɸ*_cr_, vol.%	*σ*_filler_, S/m	*t*
GNP/L285	2.10	3.7 × 10^5^	3.66
GNP (UV/O_3_)/L285	1.60	5.0 × 10^6^	4.44

It is well known that *t* = 2 for an ideal three-dimensional system. As it can be seen in Table 1, the values of critical indexes of investigated polymer CMs appear to be about 1.8–2.2 times greater compared to the theoretical value. Such behavior of critical index *t* also occurs in other conductive mixtures of PANI-PETG [18] and PANI-CA [19]. The values of the critical index, presented in Table 1 for the studied CMs, are higher than those given in the classical theory of percolation, because Eq. 1 does not take into account the specific features of the structure—filler morphology, filler interaction with the matrix, the presence of contact phenomena on the boundary particle-particle, and technological conditions of composite fabrication—which strongly influence the spatial distribution of conductive particles [20–24]. As shown in [25], the critical parameter *t* may lie in a range of 1 < *t* < 6.27 for different types of nanocarbon fillers. This difference in value can be explained also by the statistical distribution of the filler in the polymer matrix [26–28]. In addition, high value of index *t* may be attributed to the extreme geometry of conductive filler particles and indicates the presence of different mechanisms of electric transport in composites.

Tunnel conductivity between the anisometric filler particles in the real composite, which are covered with a thin layer of polymer, can be a major mechanism of electric transport in CMs at concentrations in the vicinity of the percolation threshold [29] and can lead to not universal values of critical index *t*, as well as to increase the width of the percolation transition.

As can be seen from Table 1, the setting *σ*_filler_ may differ from their own specific conductivity of the filler, which as shown in [30] is associated with both the conductive grid spatial structure of the filler particles, so the nature of the filler particles contacting each other.

So, you can see that the classical percolation theory cannot accurately describe the behavior of the concentration curves for CM nanocarbon-epoxy resin. In the classical model of percolation, power dependence of conductivity on the filler content essentially reflects only the increase in the number of conductive chains N^*^_chain_in_CM_. Therefore, to describe the behavior of electrical characteristics depending on concentration, temperature, etc., a model of effective electrical conductivity, based on consideration of the value of contact resistance between the conductive filler particles *R*_*к*_ has been proposed in [31].

According to this model, the electrical resistance of CMs with disk-shaped particles (GNP and GNP (UV/O_3_)) can be estimated from the following relation [31]:

where *F* is the packing factor (for GNP (GNP (UV/O_3_)) *F* = 0.05); *r*_GNP_ is the electric resistance of disk-shaped filler particle $\left(r=\rho \cdot \frac{d}{d\cdot h}=\frac{\rho }{h}\right);$ therefore, *z* = *h*—for GNPs, where *h* and *d* are the thickness and diameter of graphite disk-shaped particle, respectively; *R*_*к*_ is the electric resistance of single contact between the filler particles; N^*^_chain_in_CM_ is the number of connected in parallel GNP chains that conduct electric current and consequently, it is proportional to the number of particles, which take part in electric transport; and *γ* is a factor, which varies from 1 to 2. Electric resistance of chain from GNPs *R*_chain_with_GNP_ is proportional to the quantity of filler particles in one chain *N*_GNP_in_chain_$\left(N=\frac{b\left(1\ \mathrm{cm}\right)\cdot \gamma }{d}=\frac{\gamma }{d}\right),$ where *b* is the sample length (1 cm).

This model takes into account not only the critical concentration, the packing factor, and the electrical resistivity of filler but the morphology of filler particles, namely its aspect ratio, defined as *d/h* for GNPs. The aspect ratio of filler particles strongly influences on the formation of conductive chain and percolation threshold in filled CMs.

**Fig. 4:**
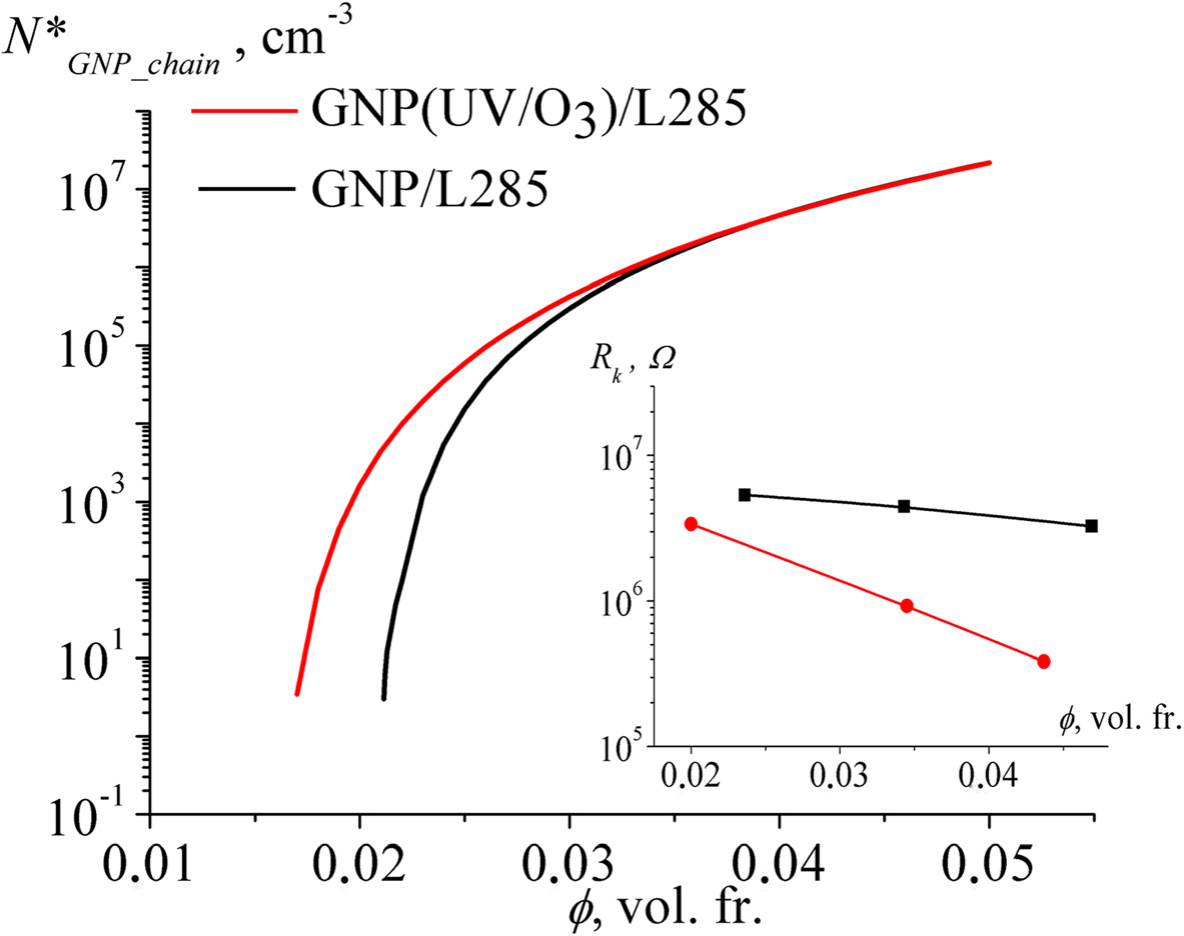
The number of conductive nanocarbon chains *N* ^*^_chain_in_CM_ and contact resistance *R*_*k*_ (in *inset*) of studied CMs, which are calculated by using Eq. (2)

Within the framework of the model of effective electrical resistivity, the values of the number of conductive nanocarbon chains N^*^_chain_in_CM_ and contact resistance *R*_*k*_ were calculated and reported in Fig. 4.

Figure 4 shows that the number of conductive chain N^*^_chain_in_CM_ increases with increasing concentration of filler in the polymer matrix from 3.11 × 10^3^ cm^−3^ (for 0.0236 vol. fr. GNP) to 1.40 × 10^7^ cm^−3^ (for 0.0469 vol. fr. GNP). At the same time, the value of contact resistance *R*_*k*_ decreases with increasing concentration of filler in the polymer matrix due to the decrease of the thickness of polymer layer between the filler particles with increasing GNP content in polymer matrix and increase of probability for formation of new conductive chains.

Let us consider the calculated values of the number of conductive chains N^*^_chain_in_CM_ and contact resistance *R*_*k*_ for CMs with UV/O_3_-treated GNPs.

As shown in Fig. 4, the number of conductive chains N^*^_chain_in_CM_ for CM GNP (UV/O_3_)/L285 also has a growing dependence on the filler concentration and significantly exceeds the values of N^*^_chain_in_CM_ in CM GNP/L285 for the same content of GNPs. The value of contact resistance *R*_*k*_ decreases with increasing concentration of nanocarbon filler, but for GNP (UV/O_3_)/L285 composites, the sharp slope of the *R*_*k*_ (*ϕ*) curve and lower value contact resistance *R*_*k*_ are observed as compared with CM GNP/L285 with the same filler content.

This change in the value of contact resistance *R*_*k*_ may be mainly due to the changing of the nature of the contact between the particles of the filler on the interface of carbon-polymer.

As it was shown, UV/O_3_ treatment of nanocarbon particles leads to additional functionalization of GNPs that improve their dispersion because of the electrostatic repulsion of the functional groups on the GNPs surface. Besides, it was revealed [17] that the removal of organic surface contaminants (pollution) and weak bonds from the graphite surface through the UV/ozone treatment may have an ameliorating effect on the formation of stronger conductive network. The organic contaminant and weak bonds may possess an electrically insulating character as the electron scatter. But their removal leads to a reduction in contact resistance *R*_*k*_ between the nanocarbon particles and the average number of conductive chains N^*^_chain_in_CM_ in CM increases.

### The Temperature Dependences of Electric Resistance CMs with GNPs and GNP (UV/O_3_)

**Fig. 5:**
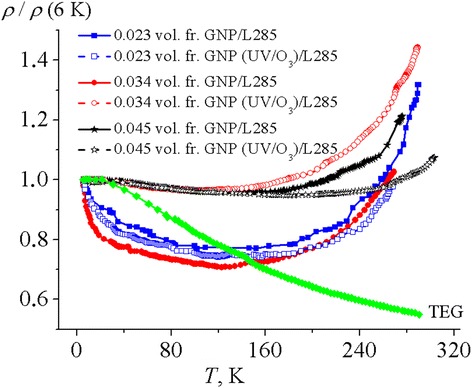
Thermal dependences of the electrical resistivity for CMs with different concentrations of GNPs before and after UV/O_3_ treatment and for pressed TEG powder

The temperature dependences of the electrical resistivity of composites based on GNP and GNP (UV/O_3_) were investigated in the temperature range 6–300 K. As it is seen from Fig. 5, the minimum on the temperature dependence of electrical resistivity of CMs is observed. The value of this minimum decreases with the increase of GNP concentration. The most clearly expressed temperature dependence of the electric resistance is observed for CM GNP/L285 with GNP concentrations of 0.023 and 0.034 vol. fr. and for CM GNP (UV/O_3_)/L285, only with 0.023 vol. fr. of GNP (UV/O_3_). This minimum of resistivity is observed for the three composites almost at the same temperature near 130 K.

Investigation of temperature dependences of electrical resistivity showed that the studied composites are characterized by negative and positive temperature coefficients of resistance (TCR): $\mathrm{T}\mathrm{C}{\mathrm{R}}_{\mathrm{CM}}=\frac{1}{R}\cdot \frac{dR}{dT}.$

For disk-shaped particles (CMs with GNPs), which form a chain structure in GNP/L285, there is a combination of negative TCR and positive TCR after a certain inflection temperature *T*_*m*_. For the composite GNP (UV/O_3_)/L285 (see Fig. 5), a gradual weakening of the temperature dependence and decrease of the value of the electrical resistivity are observed with the increase of the GNP (UV/O_3_) content in CMs. For CM GNP (UV/O_3_)/L285, the minimum of curve *ρ*/*ρ* (6 K) is shifted to more high temperatures compared with CM GNP/L285.

We also observed a decrease of resistivity values in the whole range of temperatures that may be caused by functionalizing of GNP particles, thereby increasing the degree of dispersion and uniform distribution of GNP particles in a polymer matrix and reduce the contact resistance *R*_*k*_ between the GNP particles.

According to Eq. (2), temperature dependence of electrical resistivity of CM with nanocarbon filler of optional shape is defined by temperature changes of the following three parameters [22]:

- The electrical resistivity of disk-shaped filler particle *r*_GNP_—the electrical resistivity of graphite particles decreases due to the increase of the charge carrier concentration (electrons and holes) with temperature raising under dominated temperature-independent charge carriers scattering on crystallite grain boundaries, thus TCR is negative and, consequently, *R*_CM_ decreases when the temperature increases.
- Contact resistance between the conductive filler particles *R*_*к*_.
- The number of conductive nanocarbon chains N^*^_chain_in_CM_.

Depending on which one of these temperature-dependent processes is primary, we have a positive or negative value of TCR.

We know that the average value of contact resistance between the filler particles *R*_*k*_, defined as the sum of electric resistance through direct contacts *R*_dir_, and the tunneling resistance *R*_tun_ [31] is

4 $$ {R}_{\mathrm{dir}}\left(\mathrm{T}\right)\propto {\rho}_f $$

5 $$ {R}_{\mathrm{tun}}(T)={R}_0\cdot \exp \left(\frac{T_1}{T_0+T}\right)\cdot \exp \left(A\cdot \delta (T)\right), $$

where *ρ*_*f*_ is the electrical resistivity of nanocarbon filler, the thickness of the polymer layer *δ* increases during the thermal expansion of the polymer matrix by law *δ*(*T*) = *δ*_0_⋅(1 + *α*_*T*(*p*)_*T*) (coefficient of thermal expansion *α*_*T*(*p*)_≈1.4 × 10^−4^ 1/K for epoxy resin; *α*_*T*(*f*)_≈2.8 × 10^−5^ 1/K for natural graphite), which increases the tunneling resistance *R*_tun_; *R*_0_, *T*_1_, *T*_0_, and *A*—independent of the temperature settings, and actually multiplier exp (*T*_1_/(*T*_0_ + *T*)) determine the temperature dependence of the electric resistance due to the change of the electrical conductivity of the carbon material and multiplier exp (*A*⋅*δ*(*T*))—the temperature dependence of the electric resistance, due to the nature of the filler particles contact each other (contact area *a*, the gap *δ* between the particles of filler).

As noted in [31], tunneling mechanism is the most famous to interpret conductivity in CM nanocarbon-epoxy resin near the percolation threshold. The analysis showed that the temperature dependence of the resistivity in CMs with a chain conductive structure (GNP/L285 and GNP (UV/O_3_)/L285) with filler concentrations of 0.023, 0.034, and 0.045 vol. fr. can be described within the model of effective electrical conductivity (Eq. (2)).

Change of the electric resistance at low temperatures from 6 to 130 K is equal to 10–30 % for samples GNP/L285 and GNP (UV/O_3_)/L285 with a filler concentration of 0.045 vol. fr., which correlates with the temperature dependence of the resistivity of the carbon filler (see Fig. 5). This makes it possible to believe that the main contribution to the temperature dependence of the resistivity at low temperatures in CMs is the temperature dependence of electric resistance changes by direct contact *R*_dir_ (see Eq. (4)). In the case of direct contact between the carbon particles, contact resistance between particles *R*_*k*_ depends on the electrical resistivity of nanocarbon and the contact area *a* [31]: ${R}_k=\frac{\rho_f}{2\cdot a}$ at *a* > > *l*_*pl*_ (Holm contact) and ${R}_k=\frac{\rho_f}{2\cdot a}\cdot \left(\frac{l_{pl}}{2\cdot a}\right)$ at *a* < < *l*_*pl*_ (Sharvin contact), where *a* is contact spot radius, *l*_*pl*_ is the effective path length of the charge carriers.

When the temperature rises above 130 K, the observed changes in contact resistance *R*_*k*_ are associated with the ratio between the coefficients of linear thermal expansion for graphite *α*_Gr_ and polymer *α*_P_. If *α*_P_ *> α*_Gr_, then with increasing temperature, i.e., heating decreases the contact area (decreasing radius of the contact spot *a*), as well as the direct contact between individual particles is broken and the thickness of the polymer layer *δ* increases. This leads to the increase of the tunneling resistance *R*_tun_, which exceeds reducing of direct electrical contact *R*_dir_ due to the growth in the electrical conductivity of the material (GNPs) and positive temperature coefficient of resistance (TCR) is observed (Fig. 5).

For composites GNP/L285 with a concentration of 0.023, 0.034, and 0.045 vol. fr., GNP contribution of tunneling mechanism increases, which leads to greater dependence of contact resistance *R*_*k*_ and, therefore, the resistance of the composite *R*_CM_ at low temperatures.

In CMs with GNP (UV/O_3_), a strong decrease of the total contact resistance *R*_*k*_ is observed (see Fig. 4). Beginning from the filler concentration 0.034 vol. fr. GNP (UV/O_3_) in the composite GNP (UV/O_3_)/L285, the role of direct contacts *R*_dir_ in the contact resistance between the particles *R*_*k*_ increases, so that the particles are tightly next to each other, and during thermal expansion of the polymer L285 at the increase of temperature, the polymer layer slightly increases that does not influence on the number of conductive chains N^*^_chain_in_CM_. Effect of change in the distance between particles *δ* when the temperature rises through a factor exp (*A*⋅*δ*(*T*)) in such composites occurs at much higher temperatures.

### The Frequency Dependences of Electric Conductivity CMs with GNPs and GNP (UV/O_3_)

In order to verify the correctness of our reasoning, the electrical conductivity in the range of high frequency of electromagnetic radiation has been studied.

**Fig. 6:**
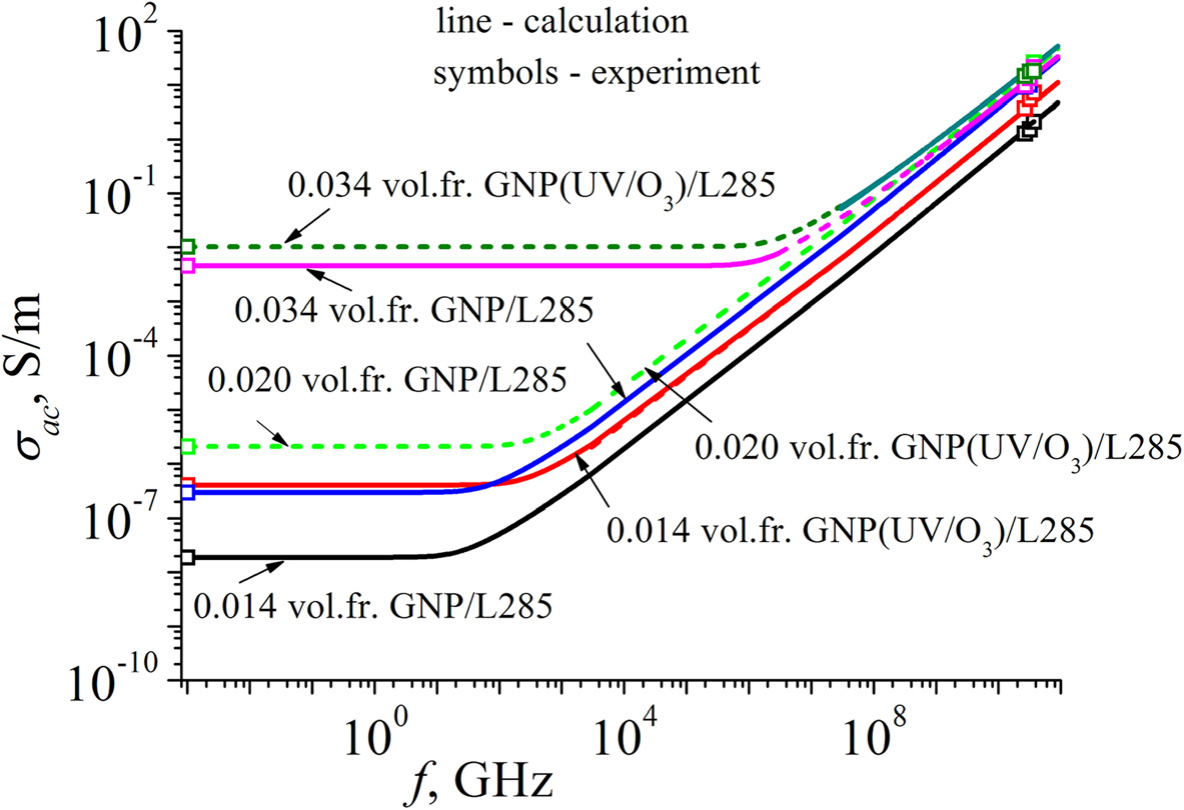
Frequency dependences of the electric conductivity of CMs with different concentrations of GNPs before and after UV/O_3_ treatment

Using the experimentally measured values of *σ*_*dc*_, *ɛ*^′^_*r*_, *tgδ* = *ɛ*^′′^/*ɛ*^′^ for high frequency and the ratio *ɛ*^′′^_*r*_ = *σ*_*ac*_/(*ɛ*_0_*ω*), the frequency dependence of electrical conductivity was reconstructed based on the equation *ɛ*^′′^_*r*_ = *σ*_*ac*_/(*ɛ*_0_*ω*). The corresponding data are presented in Fig. 6. The significant increase of electrical conductivity observed in the range of 25.5–37.5 GHz as compared with DC conductivity proves the realization of tunneling mechanism of conductivity.

When the frequency rises above a certain critical frequency *f*_*c*_, the increase of the value of the electrical conductivity has been observed. It occurs due to the additional contribution of the electrical conductivity related to the increased probability of skipping (or tunneling) of charge carriers between the filler particles. As a result, the average displacement of charge carriers decreases with increasing frequency, and the electrical conductivity behaves in accordance with the law *σ*_*ac*_~*f*^0.8^ after reaching a certain critical frequency *f*_*c*_.

It was found that the values of critical frequency of composites with low concentrations of filler (up to a critical concentration) is shifted to higher frequencies after the UV treatment of filler (we compared the composites with the same filler content).

## Conclusions

IR spectra showed that ultrasonic dispersion of expanded graphite in acetone leads to the formation of functionalized graphite nanoplatelets. It was found that ultraviolet treatment of graphite nanoplatelets during 20 min leads to a redistribution of the relative intensities of the bands between carboxyl, ketone, lactone, and carbonyl groups.

The concentration dependences of the electrical conductivity and temperature dependences of the electric resistance of the nanocomposites with GNPs without and with 20-min UV/O_3_ exposure were investigated experimentally. It is determined that the percolation threshold decreases to 0.005 vol. fr. for the nanocomposites with UV/O_3_-treated filler, and UV/O_3_ exposure of the filler improved significantly the electrical conductivity of the nanocomposites at the same GNP content.

The improvement in conductance and reduced percolation threshold is discussed within the model of the effective electrical resistivity which takes into account the morphology of the carbon filler, its intrinsic electrical conductivity, and the electrical contact resistance between particles in the formed filler chains. The calculations showed that the amount of conductive chains has increased significantly, while the value of the contact resistance between the particles of nanocarbon is more sharply decreased with increasing concentration of filler for composites with UV-treated graphite nanoplatelets. This is possible due to the effect of surface “cleaning” of the filler particles (namely due to the redistribution of the relative intensities of the bands between the carboxyl, ketone, lactone, and carbonyl groups). As a result, we have an improvement of the dispersion of the filler in the polymer matrix and new conductive chains are formed in the composites.

In the study of temperature dependences of electrical resistivity, it was found that temperature inflection has shifted toward higher temperatures in composites with GNP (UV/O_3_). This indicates that there is a greater influence of direct contacts between the filler particles and it is not contrary to the model of the effective electrical resistivity. On the other hand, the contribution of tunnel conductivity is proved by significant increase of electrical conductivity in the microwave range. If graphite nanoplatelets are subjected to UV treatment for 20 min before adding to the polymer, it leads to improved electrical characteristics and reduces the temperature dependence of the electrical resistivity in these composites.
